# Solution-Focused Brief Intervention for Substance Use: Protocol for a Multisite Randomized Controlled Trial

**DOI:** 10.2196/75628

**Published:** 2025-11-11

**Authors:** Karla González-Suitt, Alvaro Vergés, Rodrigo Portilla Huidobro, Bárbara Donoso Vargas

**Affiliations:** 1 Escuela de Trabajo Social Facultad de Ciencias Sociales Pontificia Universidad Católica de Chile Santiago Chile; 2 Escuela de Psicología Universidad de Los Andes, Chile Santiago Chile

**Keywords:** brief psychosocial interventions, clinical social work, primary health care, solution-focused brief therapy, substance use

## Abstract

**Background:**

Substance use is a high-impact biopsychosocial problem in Chile, where 21% of adults have experienced a severe alcohol use episode. In the past year, the prevalence rates were 12.5% for marijuana, 0.8% for cocaine, and 0.4% for cocaine base paste. Cannabis prevalence in Chile is higher than the global (4.2%) and South American (3.58%) averages. Cocaine prevalence in Chile is lower than in South America (1.62%) but higher than the global average (0.42%). No international reports are available for cocaine base paste. Mental health and substance use programs in Chilean primary care involve psychologists and social workers. Solution-focused brief interventions (SFBIs) are based on solution-focused brief therapy, a strengths-based and person-centered approach in which practitioners adopt a stance of “not being the expert,” respecting clients’ needs and perspectives.

**Objective:**

This study aims to determine whether the SFBI implemented by psychosocial teams (psychologists and social workers) for individuals with alcohol and other drug use in primary health care centers leads to better outcomes than usual care.

**Methods:**

We will conduct a randomized controlled clinical trial (ClinicalTrials.gov registration pending) comparing a 3-session SFBI (experimental group) with a single session of brief counselling as usual care (control group) in primary care. Interventions will be delivered in person by a psychologist or social worker. A total of 320 participants are expected to be recruited during preventive routine checkups using the Alcohol, Smoking, and Substance Involvement Screening Test. Participants reporting intermediate- to high-risk substance use on this screening tool will be randomly assigned to each group. Research assistants will administer instruments at baseline and at 3-, 6-, and 9-month follow-ups and will be blinded to the assigned treatments. The primary outcome assessed will be substance use patterns, while secondary outcomes include background information, depressive symptoms, anxiety symptoms, and motivation for treatment. Statistical analyses, including *t* tests, ANOVA, and Fisher exact tests will be conducted depending on variable type and normality. A qualitative component to assess acceptability and pertinence will include focus groups with participants and practitioners, followed by a content analysis.

**Results:**

Funding for this study started in April 2024. As of the submission date of this protocol, 55 practitioners from 9 primary health care centers have been trained in SFBIs. Recruitment began in February 2025, with 73 participants enrolled and 23 who dropped out. Recruitment will continue until December 2026. No analyses have been conducted to date. Findings are expected to be published during the second half of 2028.

**Conclusions:**

This study strengthens primary care by integrating targeted psychosocial interventions for substance use into existing programs, thereby enhancing real-world applicability. If effective, the intervention could be adopted into routine care and inform public policy on mental health and substance use.

**International Registered Report Identifier (IRRID):**

DERR1-10.2196/75628

## Introduction

### Overview

Substance use is a high-impact biopsychosocial problem in Chile. Chile has one of the highest levels of alcohol consumption per capita on the continent, with rates of 15% for men and 3.7% for women [1]. In addition, 21% of the population have experienced a severe alcohol episode, compared with the global average of 18% [1]. These high levels of alcohol consumption are associated with health indicators, including cirrhosis prevalence and alcohol-related causes of death [2]. Furthermore, illicit drug use in Chile showed a prevalence of 12.5% in the past year and 12.7% in the past month for marijuana (18.1% for men and 7.4% for women), 0.76% in the past year and 1% in the past month for cocaine (1.8% for men and 0.3% for women), and 0.4% in the past month for cocaine base paste (0.7% for men and 0.2% for women) [3,4]. The rates of cannabis use in Chile are concerning when compared with the global and South American prevalence. The global prevalence of marijuana is 4.12%, and in South America it is 3.58%. Cocaine prevalence is lower in Chile than in the rest of South America (1.62%) but remains higher than the global prevalence (0.42%) [5]. No international reports are available for cocaine base paste. In terms of age groups, marijuana and cocaine use is more common among individuals aged 19-25 years, whereas cocaine base paste is more prevalent among individuals aged 26-34 years [3]. Drug-related deaths have tripled in the Americas and are among the top 10 causes of disability-adjusted life years [6].

### Psychosocial Variables

Alcohol and drug use are associated with various psychosocial indicators, including domestic violence, child abuse, and anxiety-depressive disorders [7-10]. From a social perspective, the highest prevalence of alcohol and substance use disorders, including marijuana, cocaine, and cocaine base paste in Chile, is observed among populations with lower socioeconomic status [2]. In terms of the incidence of alcohol-related crime, 53% of crimes were attributed to alcohol use, and 52.9% of those arrested for drugs, crime, or domestic violence met the criteria for alcohol use disorders. Regarding drug use, a recent study showed that among individuals who have been arrested due to high social connotation crimes, family violence, and drug-related issues, urine tests were applied to 141 individuals (74.7% and 25.3% women); the results showed that 65% has consumed at least one substance, the most common being marijuana (58%), followed by cocaine (30%) [11]. Although, no data were reported regarding cocaine base paste, the same study reported that the prevalence of substance use during the past year was marijuana (48.4%), cocaine (21%), and cocaine base paste (11.4%) [11]. Finally, being a victim of theft, sexual abuse, or any harassment is associated with a higher likelihood of reporting higher quantity and frequency of alcohol consumption [12]. All these dimensions intersect with areas where the intervention of psychosocial teams in primary health care (PHC) centers plays a role in prevention, diagnosis, treatment referral, and follow-up.

### Chilean Public Policy for Substance Use

The range of treatment options for substance use in Chile includes various programs to address different patterns of use and diagnoses. In this context, PHC centers play a fundamental role. Within the framework of the National Mental Health Plan (Plan Nacional de Salud Mental) 2017-2025, a series of programs have been established for the benefit of community mental health [13]. These include the Psychosocial Support Program (Programa de Apoyo Psicosocial) in PHC, the Prevention and Treatment Program for Alcohol and Drug Use and/or Dependence (Programa de Prevención y tratamiento del Consumo y/o Dependencia de Alcohol y Drogas) in primary care, as well as the Explicit Health Guarantees Law (Garantías Explicitas en Salud), which since 2007 includes care for individuals under 20 years of age with harmful alcohol and drug use. Additionally, the Detection, Intervention, and Referral Program for Alcohol, Drug, and Tobacco Use (Programa Detección, Intervención, y Referencia de Alcohol, Drogas y Tabaco) is based on brief interventions aimed at reducing substance use and covers 150 communes throughout the country [13].

### Brief Interventions

It is worth noting that the duration and intensity of brief intervention programs for substance use and their effectiveness have been widely discussed in scientific literature. The Center for Substance Abuse Treatment from the United States asserts that most individuals in substance use treatment drop out before completing the program and recommends the incorporation of brief therapy techniques when offering treatment [14]. Furthermore, it has been suggested to provide a brief intervention for all individuals seeking treatment before referring them to long-term modalities [14]. In this context, brief interventions are described as a therapeutic relationship in which a professional provides one or more individuals with therapeutic assistance to achieve changes or goals to improve various aspects of the person’s life in the short term [15-18]. Interdisciplinary and collaborative work in PHC has shown positive results in addressing substance use. However, such interventions tend to be costly and more intensive than what is typically feasible to provide at this level of care, which may include psychiatrists, nurses, physicians, psychologists, and social workers. Moreover, their outcomes are similar to brief interventions [19]. The psychosocial approach to substance uses in PHC involves understanding that the problem of substance use is related to social determinants of health, as well as internal aspects of the individual, such as self-efficacy, coping strategies, motivation, and commitment [20,21].

In Chile, mental health and substance use programs in primary care involve psychologists and social workers, which often leads to working in pairs and, at times, overlapping daily intervention tasks. However, both professions have different focuses in their approach. While psychologists are often trained in the individual’s intrapsychic processes, social workers are trained to understand the context, family, and social factors or determinants surrounding the individual. The interdisciplinary approach, which involves collaborative work, mutual learning, and overlapping roles [22], is common in primary care due to the person-centered model promoted by the comprehensive health-care model [23].

A study conducted in Chile found no significant difference in substance use between a single-session brief intervention provided by social workers or psychologists, following the Feedback, Responsibility, Advice, Menu Options, Empathy, and Self-Efficacy approach, and the delivery of a pamphlet [24]. Despite these findings, this type of brief intervention is currently applied in Chilean PHC when individuals are detected with a moderate risk of substance use. In this context, the National Mental Health Plan stated the need to develop more outcome studies that address the needs and particularities of the Chilean health system and population [13].

### Solution-Focused Brief Interventions

Solution-focused brief interventions (SFBIs) are based on solution-focused brief therapy: a strengths-based and person-centered approach developed by clinical social workers in the United States that draws from systemic approaches and brief family therapy. Nevertheless, it is applicable to psychologists as well as social workers with clinical training, as it seeks to accompany others in processes of change. One notable aspect of this model is its cultural adaptability, as it approaches individuals participating in an intervention from a stance of “not being the expert” and respects their needs and perspectives [25]. An SFBI considers both individual aspects and interactions with significant others and the context to which individuals belong. Therefore, it is presumed to be more suitable for Latin populations, where close relationships and family ties are highly valued [26,27]. This is different from models such as Motivational Enhancement Therapy (Motivational Interviewing Treatment Context) [28], which focuses on enhancing individuals’ intrinsic motivation [29] without considering relational aspects.

In an SFBI, the process of change occurs in conversations about solutions. These conversations aim to co-construct meanings, identify exceptions to the problem, develop a preferred future (based on a miracle or a hypothetical situation where the problem no longer exists), and explore how the person or family has achieved those exceptions in the past to repeat them in the future, thereby, making the desired future possible [25]. A literature review regarding SFBIs with substance users has shown promising results in reducing substance use and psychosocial problems associated with it [30]. Our team has previously developed a linguistic adaptation of SFBIs [31], providing methodological support for its applicability to the Chilean population receiving primary care. After that study, 2 pilot implementations were conducted to examine the feasibility of the model by social workers in primary care [32,33]. The results of the pilot studies showed positive trends in terms of reducing days of abstinence, average use, and maximum number of drinks on a single occasion; decreasing alcohol-related consequences and depressive symptoms, and improving participants’ subjective well-being [32,33]. Given that (1) previous research has not shown conclusive evidence of a single-session brief intervention on reducing substance use patterns [24], (2) SFBIs have shown promising results in pilot studies with alcohol use [32,33], (3) there is an urgent need for evidence-based interventions in Chilean public PHC [13], and (4) current usual care in PHC allows for the introduction of a new methodology to be compared, conducting a randomized controlled trial of SFBIs adapted to Chilean culture and language and implemented by psychosocial teams, compared with usual brief interventions, could make a substantial contribution to the available knowledge on brief interventions for substance and the future implementation of public mental health policy addressing substance use in Chilean PHC.

### Objectives

The general objective of this study is to determine whether the SFBI implemented by psychosocial teams (psychologists and social workers) in individuals with alcohol and other drug use in PHC leads to better outcomes than usual care. Specifically, the study aims to determine whether the SFBI reduces substance use patterns and the risk level of substance use among participants at the 3-month, 6-month, and 9-month follow-ups post baseline. It also aims to determine whether the intervention reduces the consequences of substance use and improves mental health symptoms at these follow-up points. In addition, the study seeks to determine whether participants receiving the SFBI improve their substance use patterns, substance use risk level, consequences of substance use, and mental health symptoms as compared to those receiving a minimal intervention. Finally, the study explores whether psychosocial professionals implementing the SFBI, as well as the individuals who participated in the intervention group, find the model acceptable and relevant to primary care.

### Hypotheses

This study has the following hypotheses. First, participants who receive the SFBI will decrease their substance use patterns and risk-level use of alcohol, marijuana, cocaine, and cocaine base paste. Second, participants receiving the SFBI will report positive changes in the consequences associated with alcohol, marijuana, cocaine, and cocaine base paste use, as well as improvements in mental health symptoms between baseline and follow-ups. Third, when comparing outcomes between the intervention group and the usual care group, it is expected that participants in the intervention group will show greater reductions in substance use, consequences of use, and mental health symptoms than those receiving usual care. Finally, both participants and professionals involved in the intervention group will report experiences indicating that the model is acceptable and relevant for implementation in primary care.

## Methods

### Ethical Considerations

This protocol has been approved by the Research Ethics Committee of the Pontificia Universidad Católica de Chile (ID 230619010; May 30, 2024), the Research Ethics Committee of the Local Health Services at the Metropolitan South-East area (September 12, 2024), and the Valparaíso–San Antonio area (N°23/2024; December 11, 2024). This protocol has been registered in ClinicalTrials.gov (registration pending). However, it was previously registered in Open Science Framework Registries (osf.io/875pj) on November 19, 2024. The protocol presented in this study is the second version, approved by the aforementioned committees. All participants (patients and professionals) signed an informed consent document and can choose to stop participating in the study at any time without consequence for their relationship with the primary health clinic or the university.

Confidentiality and privacy of participants will be protected. Psychosocial professionals’ information is stored in the database to maintain contact with each of them during this study. This data will not be shared with anyone outside the study. Patients’ identifiable information will be stored in a key-protected folder, to which only the research coordinator will have access. An alphanumeric code will be generated automatically once the patient’s information is entered into the REDCap (Research Electronic Data Capture; Vanderbilt University) platform, where the instruments are embedded and data from each patient are stored and remain deidentified. Once the data collection phase is complete, the deidentified database will be downloaded and securely stored by the principal investigator in a password-protected digital folder for future research purposes. The deidentified database can be requested from the first author. The research team, professionals, and any research assistants hired to perform data tabulation or analysis tasks related to this study’s content will be trained to maintain confidentiality and will sign a confidentiality agreement. They will not possess printed or digital copies of the data.

Psychosocial professionals and patients participating in this study will be recorded on audio during randomly selected sessions within the first 10 months of SFBI implementation (5 sessions per professional). This data and information will be carefully stored and managed by the principal investigator; the names of recordings will be coded, and only an expert in SFBIs will listen to each recording to assess the practitioners’ fidelity to the model. The content regarding patients’ private lives will not be the focus of these analyses. Recordings of the sessions between psychosocial professionals and patients will be stored for 3 years in a digital folder owned by the principal investigator, protected with a secure password. Participants will be informed through the informed consent that their sessions may be recorded in audio, and they will be reminded of this if their session is recorded.

Patients will receive compensation each time they respond to the questionnaires. The compensation will consist of a grocery store gift card equivalent to US $5 at baseline, US $6 at the 3-month follow-up, US $7 at the 6-month follow-up, and US $8 at the 9-month follow-up. The gift cards are redeemable for food and are intended to compensate participants for the time spent answering the questionnaires.

### Study Design

The design of this study corresponds to a superiority randomized controlled clinical trial, conducted in public primary care clinics, to evaluate the effect of an SFBI and compare it with usual care, consisting of brief counseling and an educational brochure. The experimental and control groups will consist of primary care patients who report intermediate- to high-risk substance use and will be randomly assigned to each group. In addition, the acceptability and relevance of the applied intervention will be explored among participants in the experimental group using a qualitative approach, which focuses on a deep understanding of experiences considering the subjectivity and meanings attributed to them by individuals [34], through focus groups and qualitative analysis of their content. This study is guided by the SPIRIT (Standard Protocol Items: Recommendations for Interventional Trials) checklist (Multimedia Appendix 1).

### Sample Size

The necessary sample size was calculated considering the minimum effect size found in evidence-based psychosocial interventions for substance use disorders (Cohen *d*=0.24 for polysubstance use, equivalent to Cohen *f*=0.12) [35]. The calculation was performed using the G*Power (Heinrich-Heine-Universität Düsseldorf) statistical software for sample size calculation [36], considering 80% power and a significance level of 5%. For the interaction effect between within-subject and between-subject factors in mixed ANOVA (details available in the Analysis Plan section), a sample size of 96 individuals was obtained, and after considering a 40% attrition rate [24,35] in follow-up, it increases to 160 individuals. The evaluation of the SFBI will be conducted separately by substance type (evaluating its effectiveness separately for at least alcohol, cocaine, and cocaine base paste, considering possible overlaps between them and marijuana). Thus, we plan to recruit 320 participants.

### Recruitment

Since in PHC centers the Alcohol, Smoking, and Substance Involvement Screening Test (ASSIST) is applied routinely—in preventive check-ups—to individuals aged 20-65 years, the staff members responsible for applying this test at each PHC clinic will invite individuals whose scores are equal to or higher than 11 on the ASSIST for alcohol and 4 for other substances to participate in this study. If they accept, they will be given an appointment with the research coordinator or a research assistant, who will formally invite them to participate.

The inclusion criteria are as follows: participants must have an ASSIST score of 11 or higher for alcohol and a score of 4 or higher for other substances, be able to communicate verbally with others, be willing to participate in the intervention, and be willing to complete the measurement instruments.

The exclusion criteria are as follows: participants with a severe and untreated mental illness such as schizophrenia, those not willing to participate in the intervention, those not willing to complete the measurement instruments, those who have received another brief intervention within the last year, and those currently being treated in another program for substance use will not be eligible to participate.

### Interventions for Control and Experimental Groups

For the control group, the intervention will be 1 brief counseling session and an educational brochure, provided as part of usual care in PHC. Our team has provided the practitioners participating in this study with a guideline to conduct this brief counseling based on the recommendations of the World Health Organization (WHO) [37]. These materials can be requested from the first author.

For the experimental group, the intervention will be a 3-session brief intervention based on solution-focused practices, implemented by trained social workers or psychologists, who will receive 24 hours of training provided by this study. Our team has designed a manual with the goals and structure of each session, main interventions, and examples of application in different situations. The manual can be requested from the first author. The fidelity of this intervention will be monitored through the application of the Fidelity Instrument [38] to 5 audio recordings of sessions per practitioner participating in the program. This adapted instrument, used in a previous study by the research team [32], contains techniques and skills that professionals should implement in SFBIs. It consists of 13 items with binary responses (1=yes and 0=no). In addition, once the recruitment of participants starts, practitioners in this study will participate every 2 weeks in a 1-hour group consultation meeting, where a master’s in clinical social work with advanced training in SFBIs will supervise the group in the application of SFBI techniques—focusing on best practices, solving questions, reviewing difficulties, and strengthening adherence of practitioners.

### Data Collection

**Figure 1:**
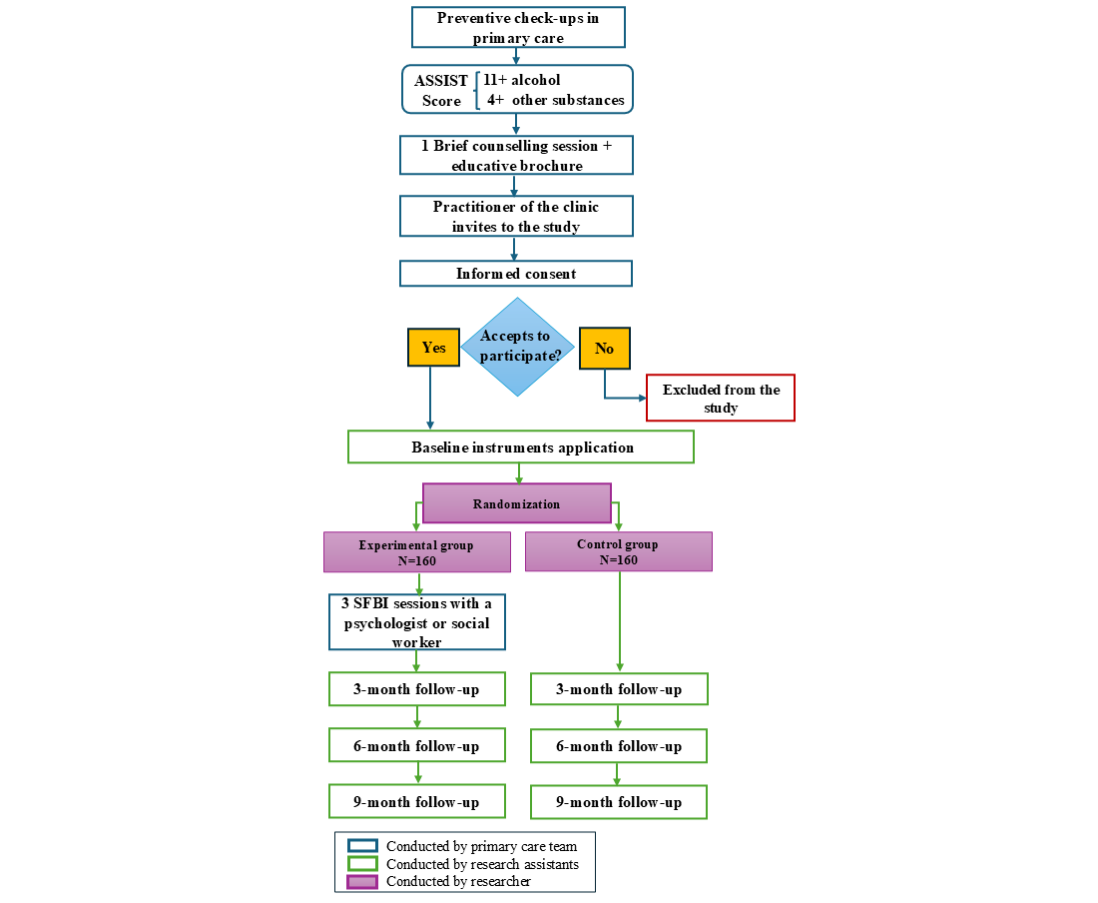
Research protocol flowchart. ASSIST: Alcohol, Smoking, and Substance Involvement Screening Test; SFBI: solution-focused brief intervention.

The protocol for participants’ invitations begins once the patient is eligible (Figure 1). The practitioner who detects an eligible participant in the PHC will conduct usual care, consisting of a brief counseling intervention of 10-15 minutes, focused on providing advice and an educational brochure with information about the risks of substance use. Following that, the practitioner will invite the potential participant and inform them regarding the implications of participating in this study, including the set instruments to complete, the possibility of receiving 3 SFBI sessions or only the brief counseling already provided, and the voluntary nature of their decision to participate. If the potential participant agrees to participate in the study, both participant and practitioner will read and sign the informed consent form. Neither of them will know which group the participant will be assigned to at this stage.

Once a participant has signed the informed consent form, they will be contacted by the research coordinator to complete a set of measurement instruments 4 times over a total estimated period of 9 months. Even though participants will know which group they were assigned to after completing the baseline forms, research assistants who interact directly with participants will not be aware of the assigned treatments (blinded).

Measurement instruments will be applied to assess baseline, progression, and follow-up for possible changes in outcome variables. The existence of self-selection bias is acknowledged and will be addressed by random allocation of participants to the control or intervention group [39]. The randomization process will be conducted by one of the research members (AV), using a computer-generated randomization sequence with a maximally tolerated imbalance of 3 using the big stick method. Randomization will be stratified so that each center is balanced in terms of participants assigned to each group.

### Instruments

#### Background Information

This includes age, gender, socioeconomic status, education level, employment status, county of residence, and family situation (relationship status, children, and their ages). This information is relevant for documenting and accounting for the heterogeneity or homogeneity of the study’s participants and will serve as control variables for data analysis. This instrument will be administered completely at baseline and at the 9-month follow-up and partially (employment status and county of residence) at the 3- and 6-month follow-up, to visualize changes in sociodemographic variables and the identification of protective and change factors during the process.

#### ASSIST

This 8-item questionnaire developed by the WHO, is used to detect risk in substance use [37]. It predicts low, intermediate, and high risk due to substance use. It is a relatively new instrument, recently validated in Chile and recommended by the WHO for assessing risk in alcohol and substance use. The validation in Chile [40] considers a score equal to or greater than 11 for intermediate to high-risk alcohol consumption and a score equal to or greater than 4 for intermediate to high-risk substance use. It is one of the main dependent variables of the study and will be administered at baseline and follow-ups.

#### Treatment Outcome Profile

This instrument has been validated in Chile [41] and was originally developed by the National Treatment Agency for Substance Misuse [42]. It is used to obtain comparable data and goals regarding improvements of individuals with substance use after treatment. It consists of 3 sections: substance use in the past 4 weeks, offensive behavior (social norms such as theft, drug dealing, fights, and domestic violence), and physical and psychological health, including quality of life, work, education, and housing. In total, there are 17 questions. It will be administered at baseline and follow-ups.

#### Patient Health Questionnaire-9

This self-administered 9-item instrument, used in primary care settings, corresponds to the depression module of the Primary Care Evaluation of Mental Disorders, which identifies various mental health disorders [43]. It reflects the 9 symptoms of depression from the *DSM-IV* (*Diagnostic and Statistical Manual of Mental Disorders* [Fourth Edition]). It has high convergent validity (*r*=0.73; *P*<.001) with the short version of the Beck Depression Inventory in detecting depression severity [44]. It has been validated in the Chilean population by Baader et al [45]. It will allow the observation of depressive symptoms prevalence in individuals using different types of substances at baseline and follow-ups, as well as exploring the association between them.

#### Generalized Anxiety Disorder-7 Scale

This is a self-report instrument to assess generalized anxiety disorder (GAD), based on GAD symptoms from DSM-IV. In primary care, its construct validity showed strong correlations between the Generalized Anxiety Disorder-7 (GAD-7) scale’s severity scores and worsening function on all 6 Health-Related Quality of Life scales of the Medical Outcomes Study Short-Form General Health Survey, as well as a high internal consistency (Cronbach α=0.92) [46]. This instrument was adapted for the Chilean population by Crokett et al [47]. It consists of 7 items with 4-point ordinal responses, with 0 indicating never and 3 indicating almost every day. This instrument will allow for the observation of the prevalence of anxiety symptoms in individuals who use different types of substances at baseline and follow-ups, as well as the exploration of associations between these factors.

#### Motivation and Treatment Needs Scale

This scale, developed by Joe et al [48], is part of the Client Evaluation of Self and Treatment, a comprehensive instrument consisting of 147 items grouped into 16 scales. In this study, 2 subscales will be used: Help-seeking Desire and Treatment Readiness [49]. Together, these subscales comprise 14 items with responses on a Likert-type scale, where 1=strongly disagree and 5=strongly agree.

#### Focus Group Guides

Guidelines for these focus groups, to be conducted with the psychosocial teams and participants from the intervention group, were designed specifically for this study to explore participants’ perceptions regarding the implementability of the SFBI model for substance use in PHC contexts (Multimedia Appendix 2).

The quantitative instruments (background information, including demographic data and sociofamilial situation, ASSIST, treatment outcome profile (TOP), Patient Health Questionnaire-9 (PHQ-9), GAD-7, and desire for help and treatment readiness form) will be administered in a private room at the health care center or over the phone (depending on coordination with each patient) by the research coordinator or a research assistant. The estimated time for administering the instruments is 25 minutes per set. The qualitative instruments (focus groups for practitioners and participants) will be implemented after the implementation process has concluded, and conversations will last between 60 and 90 minutes.

Following the informed consent procedures, the instruments will be administered on 4 occasions (Table 1).

**Table 1:** Outline for application of instruments per participant.

Instrument	Baseline	3-month follow-up	6-month follow-up	9-month follow-up	After finishing follow-ups
Background information	✔			✔	
TOP^a^	✔	✔	✔	✔	
PHQ-9^b^	✔	✔	✔	✔	
GAD-7^c^	✔	✔	✔	✔	
Desire for help and treatment readiness	✔				
Focus group					✔

^a^TOP: treatment outcome profile.

^b^PHQ-9: Patient Health Questionnaire-9.

^c^GAD-7: Generalized Anxiety Disorder-7.

### Safeguarding Mental Health

Regarding mental health well-being, this SFBI focuses on positive aspects and the future, minimizing the risk of causing any harm to mental health. If a patient feels distressed or uncomfortable during an intervention session, professionals trained in SFBIs will also be trained to contain and intervene in crises. This additional training, separate from SFBI training, will consist of a 2-hour training session on psychological first aid, provided by an expert hired for that purpose. Furthermore, the research team will ensure that the health clinic has a mental health protocol in place for its regular care. Additionally, the research coordinator and the principal researcher will be available if required. If, during follow-up procedures or any health assessment, a study participant is identified as being at risk and requiring a different type of clinical intervention, the health care team will notify the research team regarding the participant’s continued involvement in the study. In all cases, clinical decisions will take precedence over research participation.

### Analysis Plan

For quantitative-specific objective 3, we will use the standard *P*<.05 criterion to make inferences. Tests to detect any potential differences between individuals who received interventions from either social workers or psychologists will be conducted. The analysis approach for each specific objective is described in the following sections.

#### Specific Objective 1

The first specific objective aims to determine whether the SFBI reduces substance use patterns and risk-level substance use of participants at the 3-month, 6-month, and 9-month follow-ups (post baseline). A descriptive analysis of the recorded variables will be conducted for each participant in the experimental group, pretreatment, and at 3 months, 6 months, and 9 months post baseline. Minimum and maximum values will be reported, and the normality of their distribution will be assessed using frequency histograms and the Shapiro-Wilk normality test. Categorical variables will be described using absolute and percentage frequencies. For individuals participating in the experimental group, the following analyses will be conducted separately at each posttreatment measurement (3 months, 6 months, and 9 months): the change in frequency and quantity of consumption according to the TOP instrument will be calculated for each participant, with the mean change and its corresponding CI estimated, and a paired repeated-measures ANOVA conducted to evaluate whether there is a significant treatment effect on the average weekly consumption; if the normality assumption is not met, the nonparametric Wilcoxon signed-rank test will be used. In addition, the percentage of individuals who decrease their risk category will be calculated, and their respective CIs estimated, and, since the TOP contains categorical items, the McNemar test will be applied to evaluate whether significant changes occurred.

#### Specific Objective 2

The second specific objective aims to determine whether the SFBI reduces consequences of substance use and improves mental health symptoms of participants at the 3-month, 6-month, and 9-month follow-ups (post baseline). For individuals participating in the experimental group, the following analyses will be conducted for each postbaseline measurement (3 months, 6 months, and 9 months): the scores obtained on the PHQ-9 instrument will be calculated and described using mean, SD, median, minimum, and maximum values, and the distribution normality will be assessed using frequency histograms and the Shapiro-Wilk normality test. The change in the PHQ-9 questionnaire score and the desire for help and treatment-readiness scales will be calculated, the means with their corresponding CIs will be estimated, and a repeated-measures ANOVA will be conducted to evaluate whether there is a significant treatment effect on each score.

#### Specific Objective 3

The third specific objective aims to determine whether participants receiving the SFBI improve their substance use patterns, substance use risk level, consequences of substance use, and mental health symptoms as compared with those receiving a minimal intervention. A descriptive analysis of the recorded variables will be conducted for each participant in both experimental and control groups at baseline and at 3 months, 6 months, and 9 months post baseline. Quantitative variables will be described using mean, SD, median, minimum, and maximum values, and their distribution normality will be assessed using frequency histograms and the Shapiro-Wilk normality test. Categorical variables will be described using absolute and percentage frequencies. For each posttreatment measurement (3 months, 6 months, and 9 months), the following analyses will be conducted: the change in substance use patterns and substance use risk level (TOP) will be calculated for each participant in both experimental and control groups, and the mean with its corresponding CI will be estimated; the change in the PHQ-9 score will be calculated for individuals in both experimental and control groups, and the mean with its corresponding CI will be estimated; the change in the GAD-7 score will be calculated for individuals in both experimental and control groups, and the mean with its corresponding CI will be estimated. Baseline characteristics, including demographic background and sociofamilial situation, of participants in the control and experimental groups will be compared, with *t* tests or Wilcoxon-Mann-Whitney tests used for quantitative variables when the normality assumption is unmet, and the Fisher exact test used for categorical variables. Given that this is a superiority design, 1-tailed tests will be used for the following comparisons. The mean decrease in monthly consumption will be compared between individuals in the experimental and control groups using a mixed ANOVA, with group (experimental and control) as the between-subjects factor and time of measurement (pretreatment, 3 months, 6 months, and 9 months) as the within-subjects factor. Similarly, mean PHQ-9 scores will be compared between the two groups using a mixed ANOVA, with group (experimental and control) as the between-subjects factor and time of measurement (pretreatment, 3 months, 6 months, and 9 months) as the within-subjects factor, with the Friedman-aligned ranks test applied if the variable normality assumption is unmet. Finally, the percentage of individuals who decrease their substance use patterns and risk (TOP) will be compared between the experimental and control groups, using the Fisher exact test.

#### Specific Objective 4

The fourth specific objective aims to explore whether psychosocial professionals implementing the SFBI and the individuals who participated in the intervention group find that the model is acceptable and relevant to primary care. The transcribed focus groups will be analyzed using content analysis methodology to identify the concepts and themes in the transcribed data. Initially, the concepts appearing in the texts will be recognized and grouped into themes. According to Hsieh and Shannon [50], content analysis can be approached in two ways. The first is a deductive strategy, with predefined thematic categories established by the researchers based on the goals of the analysis or previous theories. The second is an inductive strategy, without predefined categories, as these are constructed based on the findings, maintaining conceptual openness for analysis. In this case, both strategies will be combined, using the predefined categories of acceptability and relevance of the SFBI from the perspective of professionals and individuals, while remaining open to other new categories or concepts emerging in the focus group conversations.

#### Data Exclusion and Missing Data

Analyses will be conducted including and excluding outliers and differences between these analyses will be reported. Analyses will be performed according to the intention-to-treat principle. Missing data will be handled using multiple imputation.

#### Data Monitoring

A Data Monitoring Committee has not been included in this protocol because the trial involves psychosocial interventions delivered by trained professionals (social workers and psychologists). These interventions are noninvasive and carry minimal risk to participants. Participants’ safety will be ensured through mental health safeguarding procedures and adherence to ethical guidelines throughout the study.

## Results

Funding for this study started in April 2024. As of the submission date of this protocol, 9 primary care clinics from 2 regions of Chile are participating in this study; 55 practitioners (30 social workers and 25 psychologists who work in these clinics) have been trained in a 36-hour course on the theory and practice of the SFBI model. The recruitment phase for participants started in February 2025 and will continue until December 31, 2026. To date, a total of 2542 ASSIST assessments have been reported by the clinics. A total of 82 patients have been referred to the study, 73 have been enrolled, and 23 have dropped out, leaving 50 currently enrolled (32% attrition).

## Discussion

### Study Contributions

This study aims to determine whether a 3-session SFBI demonstrates better outcomes in substance use patterns, risk level, and consequences, as well as mental health symptoms, when compared with usual care. By evaluating the effectiveness of an SFBI, this study aims to expand knowledge on psychosocial interventions, specifically in PHC, by testing a resource-focused model for addressing substance use. In this sense, this study promotes scientific development for the interdisciplinary approach to substance use interventions. A previous study on a single-session brief intervention also trained social workers and psychologists, acknowledging the relevance of brief interventions implemented by practitioners other than physicians [24]. However, studies examining brief interventions by interdisciplinary teams remain scarce.

It is known that previous studies have shown mixed results regarding the effectiveness of brief interventions on substance use in PHC [51,52] and that a previous study conducted in Chile showed no significant results after a single-session brief intervention (17 minutes on average) [24]. The studies referenced did not include a solution-focused model for the intervention; instead, they were based on other models, such as motivational interviewing and cognitive behavioral therapy. On the other hand, recent reviews on solution-focused practices found that research regarding the effects of SFBIs on substance abuse is scarce, and that even though results are promising, either for substance use or psychosocial comorbidities, more randomized controlled trials are needed to strengthen the evidence supporting this trend [30,53].

A key strength of this study lies in its targeted approach to enhancing outcomes for individuals with substance use in PHC. By integrating this specific intervention methodology within extant programmatic lines and collaborating with the teams expected to implement brief interventions [13], the study aligns closely with real-world practice. If proven effective, this innovation is highly likely to be adopted and embedded in PHC usual care. This work will generate valuable and applicable insights for primary care, influencing public policy frameworks regarding mental health and substance use [13].

This study has some limitations. Since it is a multisite study, the strategies used by the practitioners applying screening, detection, and delivering usual brief interventions might vary widely. To manage this variability, our research team standardized the flowchart, the speech for the usual brief intervention, and the speech for the invitation to the study. Thus, each practitioner participating in the recruitment process received a pack of laminated sheets to keep them visible, so that even though there are more than 50 practitioners involved, all have the same orientations for procedures. The variability of sites and practitioners participating in the implementation of SFBIs also needs to be managed—the training was standardized, each practitioner received a pocket guide of SFBIs, all the practitioners participate in a 1-hour fortnightly meeting for group technical supervision, and their recordings of SFBI sessions receive written feedback to strengthen the fidelity of interventions. Nonetheless, the diversity of sites is a strength; the study is being implemented in the real world, and, as such, insights from the implementation will bring valuable knowledge regarding how primary care works and what the real-world challenges are in implementing innovations and programs in primary care.

### Future Directions

The research team systematically documents insights by recording meeting notes, gathering practitioner feedback, and identifying barriers, misinformation, and implementation challenges that inform future research and practices [54]. Good practices are actively disseminated across teams to ensure the transferability of the implementation process. Preliminary findings from this study will be presented at academic and professional conferences. Stakeholders, including clinic directors, supervisory teams within the Health Services, the Division of Primary Health Care at the Ministry of Health, and Treatment and Preventive Divisions at the National Service for Alcohol and Drug Use Prevention and Rehabilitation, are being periodically updated on the study’s progress. The results will be disseminated through academic journals, conferences, and seminars. In addition, a dedicated symposium will be organized to present the study’s results. This event will bring together primary care practitioners, academic scholars, supervisory teams, and policymakers, fostering dialogue and reflection on the outcomes and broader implications for future research and policy implementation. An audiovisual presentation of the results will be shared through the clinics’ social media and other platforms, ensuring study participants interested in the findings have easy access. As most of the research on interventions for substance use has been conducted in high-income countries, and the evidence on the effectiveness of brief interventions is not conclusive [51,55], once findings demonstrate the effectiveness of SFBIs, future research in Chile and Latin American countries could compare the outcomes of several implementation formats, frequencies, and doses of intervention for diverse risk levels. For example, multigroup designs may be used to compare individual, family, group, and online interventions for different risk levels of substance use, including variations in frequencies and doses of intervention, building on previous research and filling current gaps.

## Appendix

Multimedia Appendix 1 SPIRIT checklist.

Multimedia Appendix 2 Focus groups guides.

Multimedia Appendix 3 Peer-review report by Agencia Nacional de Investigación y Desarrollo (National Agency of Research and Development; ANID), Ministry of Science, Technology, and Innovation, Government of Chile.

## Abbreviations

ASSIST: Alcohol, Smoking, and Substance Involvement Screening Test
DSM-IV: Diagnostic and Statistical Manual of Mental Disorders (Fourth Edition)
GAD: generalized anxiety disorder
GAD-7: Generalized Anxiety Disorder-7
PHC: primary health care
PHQ-9: Patient Health Questionnaire-9
REDCap: Research Electronic Data Capture
SFBI: solution-focused brief intervention
SPIRIT: Standard Protocol Items: Recommendations for Interventional Trials
TOP: treatment outcome profile
WHO: World Health Organization

## Data Availability

The datasets generated or analyzed during this study are available from the corresponding author on reasonable request.
