# Supplementary material for: Solution-Focused Brief Intervention for Substance Use: Protocol for a Multisite Randomized Controlled Trial
Source: JMIR Res Protoc. 2025 Nov 11;14:e75628. doi: 10.2196/75628 (PMC12648120; doi:10.2196/75628)
Supplement: Multimedia Appendix 2 [file resprot_v14i1e75628_app2.pdf]

Focus Group Guide for Clients

1. From your point of view, how do you assess the help that you received from the health provider who assisted you regarding your mental health issue in the Project? Why? (ask for examples)
2. What should be maintained regarding the style of the provider? Why? (ask for examples)
3. Please, tell us what you liked most about the conversation sessions. Please avoid commenting on private details.
4. What should be changed? Why? (Ask for examples)
5. How would you describe the help relationship that the practitioner had with you?
6. Is there anything that the therapist did that was different from other help experiences you have had in the past? Please describe (ask for examples)

Focus Group Guide for Practitioners

1. What opinion do you have regarding the training? Why? (ask for examples)
2. What would you improve from the training? Why? (ask for examples)
3. What would you maintain in the training? Why? (ask for examples)
4. What opinion do you have about the model of Solution-focused brief intervention? Why? (ask for examples)
5. What differences do you see between traditional models and/or the type of intervention that you practiced before learning the SFBI? Why? (ask for examples)
6. What aspects of the model do you believe apply to your context of intervention with individuals with substance use? Why? (ask for examples)
7. What aspects of the model do you think do not apply to your context? Why? (ask for examples)
8. What aspects do you think could be adapted? Why? (ask for examples)
9. If yoy had to choose a technique that you could use in every session, what would it be? Why? (ask for examples)
10. What aspects of the Project in general did you find most useful? Why? (ask for examples)
11. What aspects of the Project would you not replicate in another project?
12. Do you have any other ideas you might want to add?
