# Supplementary material for: Solution-Focused Brief Intervention for Substance Use: Protocol for a Multisite Randomized Controlled Trial
Source: JMIR Res Protoc. 2025 Nov 11;14:e75628. doi: 10.2196/75628 (PMC12648120; doi:10.2196/75628)
Supplement: Multimedia Appendix 3 [file resprot_v14i1e75628_app3.pdf]

ANID/Subdirección de Proyectos/N°89

Santiago, 6 de Marzo del 2024
Ref.: Proyecto Nº 1241837

Estimada Sra. KARLA GONZALEZ:

En nombre de la Agencia Nacional de Investigación y Desarrollo, me dirijo a usted para informar que ha sido aprobado su proyecto N°1241837, postulado al Concurso Nacional de Proyectos Fondecyt Regular 2024 en el Grupo de Evaluación de INTER - TRANS DISCIPLINARIO.

En la presente convocatoria, concursaron 1.934 proyectos, de los cuales 693 fueron adjudicados (35,8%).

En el Grupo de INTER - TRANS DISCIPLINARIO concursaron 148 propuestas y adjudicaron 50 (33,8%). Su proyecto obtuvo el lugar N° 26 y un puntaje de 4,020.

A continuación de esta carta, se informan las calificaciones y comentarios que recibió su proyecto y se incluye un certificado que acredita la adjudicación de éste. Asimismo, en el Sistema de Evaluación en Línea accederá a lo siguiente:

a. Presupuesto aprobado para cada año de ejecución, pudiendo redistribuir los fondos asignados anualmente, si lo estima pertinente.
b. Informe Autorizaciones-Certificaciones, si corresponde. Indica los documentos que debe presentar para dar inicio al proyecto en un plazo de tres meses contados a partir de la fecha de la presente carta (numeral 11.2.1. literal d) de las bases concursales).
c. Botón “Decisión de Ejecutar”. Al presionar debe comunicar si acepta o rechaza la adjudicación del proyecto en un plazo máximo de 10 días hábiles administrativos, a partir de la fecha de esta carta.

Respecto al proceso de Firma de Convenio y Transferencia de recursos, revise el instructivo disponible en InstructivoFirmaConvenioRegular2024.pdf y considere que, en caso de consultas o solicitudes relacionadas con la ejecución de su proyecto, éstas deben ser enviadas a través de Ayuda ANID (https://ayuda.anid.cl).

Desde ya reciba nuestras felicitaciones por este importante logro en su carrera científica.

Saluda atentamente a Ud.,

RESULTADO DEL PROCESO DE EVALUACIÓN

CALIFICACIONES OBTENIDAS POR EL PROYECTO Nº 1241837

Factores de evaluación / Ponderación	Calificación
Etapa 1:<br>Productividad del/de la Investigador/a Responsable (30%) (*)	3,6
Etapa 2:<br>Calidad, Factibilidad y Novedad Científica o Tecnológica (70%)	4,2

(*) información respecto a la calificación obtenida en este ítem, será enviada en una carta al correo electrónico informado en su postulación.

COMENTARIOS DE LA EVALUACIÓN
PROYECTO Nº 1241837

Nota:
Los comentarios que emita el Grupo de Evaluación podrá relevar aspectos que considere necesario transmitir a quienes postulan, de acuerdo con el numeral 9.5 de las bases concursales.

CALIDAD, FACTIBILIDAD Y NOVEDAD CIENTÍFICA O TECNOLÓGICA DE LA PROPUESTA

Estado del arte:
El consumo de sustancias es un problema biopsicosocial de alto impacto en Chile, donde se dan altas tasas de consumo de alcohol y drogas como marijuana, cocaína y pasta base. Las muertes asociadas al consumo de alcohol y drogas han aumentado en las Américas lo que se asocia a violencia doméstica, abuso infantil y desórdenes de ansiedad y depresión. El enfoque psicosocial comprende que el problema del consumo de sustancias está relacionado con los determinantes sociales de la salud, así como con aspectos personales, como la autoeficacia, las estrategias de afrontamiento, la motivación y el compromiso.
La gama de opciones de tratamiento para el consumo de sustancias en Chile incluye diversos programas para abordar diferentes patrones de consumo y diagnósticos donde los centros de atención primaria en salud (APS) y centros comunitarios de salud mental (COSAM) juegan un papel determinante. En el marco del Plan Nacional de Salud Mental 2017-2025, impulsado por el MINSAL (2017), se han establecido una serie de programas en beneficio de la salud mental comunitaria. La duración y la intensidad de los programas de intervención breve para el uso de sustancias y su efectividad han sido ampliamente discutidos. El Center for Substance Abuse Treatment de EE.UU. indica que la mayoría de los individuos en tratamiento por abuso de sustancias abandonan antes de completar el programa. Dentro de los disponibles, se destaca la Intervención breve basada en soluciones (Solution-Focused Brief Interventions (SFBI) como posible aproximación interdisciplinaria efectiva para población en Chile.

Para ello, este equipo de investigación propone evaluar la efectividad de una intervención breve centrada en soluciones aplicada a personas que tienen un riesgo alto o moderado de consumo de alcohol, marihuana, pasta base de cocaína y/o cocaína que acuden a los APS y COSAM de la RM y O´Higgins, con la finalidad de reducir el consumo de sustancias.
En el marco teórico se revisa muy poco de la evidencia disponible acerca del tratamiento de estos trastornos en Chile, sin que quede del todo claro por qué y con respecto a qué se evaluará una nueva intervención. La intervención se describe como interdisciplinaria pues involucra el trabajo y aproximación psicológica y de trabajo social, pero la aproximación investigativa no es claro que sea interdisciplinar en sí misma.
La IR de la propuesta publicó una adaptación lingüística de la SFBI dando sustento a esta propuesta de intervención, y se desarrolló un piloto para evaluar la viabilidad del modelo por parte de trabajadores sociales en APS.

Hipótesis y Objetivos: Las hipótesis son coherentes con el problema, aunque parecen redundantes. Las hipótesis están formuladas sin precisión estadística, como corresponde para estudios experimentales, y sin definir en comparación con qué disminuirán los valores de la variable dependiente (hipótesis 1 y 2). La hipótesis 3 es más un supuesto que una hipótesis como tal, y no tiene base empírica, es decir, no se sigue de la formulación del problema.
El objetivo general no define el efecto en qué se evaluará.
Los objetivos específicos son coherentes con el objetivo general e hipótesis.

Metodología: Se propone un estudio clínico experimental aleatorizado de doble ciego. No queda claro quienes están ciegos a la intervención (¿los pacientes y tratantes?). Los grupos experimental y de control estarán formados por pacientes de atención primaria que reportan un uso de sustancias de riesgo intermedio a alto y que serán asignados aleatoriamente a cada grupo para recibir la SFBI o una educación mínima, que consistirá en la entrega de un brochure educativo (intervención control).
Se describe que se comparará la intervención centrada en soluciones con la entrega de folletos. Participarán seis centros donde se entrenarán profesionales de los APS y COSAM (24 horas de duración) para desarrollar la intervención. Esta será certificada por la Escuela de trabajo social de la UC (Institución de la IR). Consistirá en una capacitación intensiva inicial y luego monitoreo bisemanal. Se grabarán cinco sesiones por centro/profesional al azar para analizar la fidelización de la manipulación de la variable independiente. Sin embargo, luego se señala que la intervención consistirá en 3 sesiones, lo que parece contradictorio con lo anterior.
Más allá de lo anterior, la metodología se describe detallada y de manera fundamentada y es coherente con los objetivos de la propuesta. Se incluyen consideraciones éticas y de cuidado. Los análisis se describen detalladamente en relación con cada objetivo y son adecuados.
Describen en detalle el cálculo del tamaño muestral (96 sujetos), que sube a 160 por factores de pérdida. Como la intervención será establecida por cada tipo de consumo, aumentan al doble el número de sujetos a incluir (320). Esté cálculo no queda del todo claro cómo lo hacen y cómo controlarán por el consumo múltiple de las diferentes sustancias.
La propuesta trata de un tema relevante, está planteado por un equipo con experiencia, se plantean métodos que manejan y resguardan los aspectos éticos tanto de los participantes como de los profesionales. El plan de trabajo es muy detallado, consistente, el análisis de la información es acuciosa y el equipo de investigación consolidado.

Plan de trabajo: El plan de trabajo de 4 años considera todas las acciones en detalle con temporalidad adecuada

separada en cuatrimestres.

Equipo de trabajo: el equipo es pequeño, pero muestra experiencia en el tema de la propuesta y en el ámbito de la salud mental. La IR no tiene experiencia liderando proyectos de investigación con financiamiento externo, sí interno. Un investigador cuenta con extensiva experiencia en esta línea. El tercer investigador no muestra productividad científica.
La IR y Co-I tienen experiencia en el SFBI, en adicciones y en entrenamiento de profesionales en estos temas. Han participado en importantes instancias como SENDA y manejan los instrumentos a aplicar. Tiene proyectos previos en temas de adicciones en adolescentes. Uno de los Co-I no refiere publicaciones
Aunque la IR y Co-I tiene trabajo previo en las APS, no hay cartas de apoyo.
Proponen la formación de 5 estudiantes, pero no indican si son tesistas de pre y postgrado.

Infraestructura y recursos: Los recursos son apropiados en general, aunque el entrenamiento en la intervención no se entiende por qué se paga aparte, si la IR es quien lo haría. Es excesivo el pago considerando que la IR tiene la experticia. Los costos de difusión también son elevados.

Novedad cientifica: El proyecto tiene novedad científica pues hay una brecha de conocimiento clara y es un problema de alta relevancia científica y social.
Es una propuesta que aporta a la disciplina, implementa una metodología de intervención breve que puede ser un aporte a los sistemas de seguimiento de personas con alto riesgo de adicción al alcohol y drogas.
Los investigadores tienen experiencia en la temática, y han aportado en proyectos previos en la temática. Esta propuesta, sin embargo, parece tener una magnitud e impacto mayor a lo que han realizado hasta la fecha.
Proponen colaboradores internacionales, una de ellas visitaría a la IR en el 1er año, fortaleciendo la capacitación de los profesionales en la metodología SFBI.
Se trata de un proyecto cuya intervención es psico-social peor no se aprecia interdisciplinariedad en el abordaje del estudio. Se trata de una investigación de psicología social acerca de una intervención psico-social. Este ámbito puede fortalecerse.

Agencia Nacional de Investigación y Desarrollo

Ministerio de Ciencia, Tecnología, Conocimiento e Innovación

CERTIFICADO ADJUDICACIÓN
CONCURSO FONDECYT REGULAR

6 de Marzo del 2024

Camilo Erazo Leiva, Subdirector (s), Subdirección de Proyectos de Investigación, de la Agencia Nacional de Investigación y Desarrollo, certifica que Doña KARLA PRISCILLA GONZALEZ SUITT, ha adjudicado el proyecto N°1241837 en el Concurso FONDECYT Regular 2024, titulado EFFECTIVENESS OF SOLUTION FOCUSED BRIEF INTERVENTIONS BY PSYCHOSOCIAL TEAMS ON SUBSTANCE USE DIMINISHING..

El proyecto, patrocinado por el/la PONTIFICIA UNIVERSIDAD CATOLICA DE CHILE, contempla una duración de 4 años – desde 1 de Abril del 2024 hasta el 31 de Marzo del 2028 –, y un financiamiento de $[illegible], $[illegible], $[illegible] y $[illegible] para cada año de ejecución, respectivamente.

Se extiende el presente certificado al/a la interesado/a, para los fines que estime conveniente.

Moneda nº1375,
Santiago, Chile
Tel: 56 (2) 2365 4600
www.anid.cl
@AnidInforma

Gobierno de Chile
